# A multiple correspondence analysis of necropsy findings in non-caged laying hens that died during the production period

**DOI:** 10.1016/j.psj.2026.106734

**Published:** 2026-03-03

**Authors:** Vibe P. Butler-Lund, Liza R. Nielsen, Ida C.N. Thøfner, Jens P. Christensen

**Affiliations:** a Bacteria and Viruses, Department of Veterinary and Animal Sciences, University of Copenhagen, Stigbøjlen 4, 1870 Frederiksberg C, Denmark; b Animal Health and Welfare, Department of Veterinary and Animal Sciences, University of Copenhagen, Grønnegårdsvej 8, 1870 Frederiksberg C, Denmark

**Keywords:** Laying hen, Mortality, Pathology, Multiple correspondence analysis, Cannibalism

## Abstract

Causes of normal mortality in laying hens have received relatively little scientific attention, despite health, welfare, economic and environmental implications. Targeting control and preventative measures to currently relevant flock-specific mortality causes is essential for successful outcomes in commercial flocks. Monitoring of mortality causes is a prerequisite. However, diagnostic criteria are not consistently reported and terminology varied between previous studies.

The objective of this study was to investigate associations between pathological findings in dead laying hens with an explorative approach applied to a previously obtained dataset on pathological findings in Danish laying hens from seven non-caged commercial flocks. This approach may provide new insights compared to descriptive analysis of predefined diagnoses. A dataset with pathological findings across 49 pathological variables in 1,648 laying hens was analyzed with multiple correspondence analysis to explore multidimensional associations between pathological findings in laying hens and how patterns in combinations of findings of individual hens may cluster as diagnoses or tentative causes of death.

Unlike descriptive statistics, the multivariate approach enabled us to illustrate the complexity of pathological processes. The first four dimensions of the multiple correspondence analysis were retained, accounting for 20.2% of the variance. The results indicated clustering of hens suggesting diagnoses of similar expected chronicity and general etiology (infectious versus non-infectious). The most dominant clusters corresponded to the two most common causes of mortality diagnosed on the same hens and reported in previous studies: salpingitis-peritonitis and cannibalism. In addition, the results suggested at least two different clusters of hens that died due to cannibalism (acute or prolonged course with concurrent pathologies). These may point to relevant differences in etiology and pathogenesis that should be explored in future studies. We suggested recommendations for time-efficient field necropsies and preventative and control measures to target the most common causes of mortality in laying hens. The results may be used by farmers and their advisors to improve monitoring of health and welfare of laying hen flocks in non-cage housing systems.

## Introduction

Mortality is an essential indicator of health and welfare in laying hens (Rodenburg et al., 2008), but causes of mortality have received relatively little attention in the scientific literature. Tailoring control and preventative measures to the actual mortality causes may improve effectiveness of these measures to the benefit of hen health and welfare, as well as financial return (Jones et al., 2019) and environmental footprint of egg farming (Weeks et al., 2016).

Normal, or non-outbreak related, mortality is generally understood as daily mortality in clinically healthy laying hen flocks with expected productivity (Liebhart et al., 2023). Since the 1950′s, only a few studies have investigated pathological causes of mortality in laying hens (Jordan, 1956; Grimes, 1975; Randall et al., 1977; Abrahamsson et al., 1998; Tablante et al., 2000; Weitzenbürger et al., 2005; Shini et al., 2006; Fossum et al., 2009; Stokholm et al., 2010; Kajlich et al., 2016; Fulton, 2017, 2019; Zloch et al., 2018; Gretarsson et al., 2023; Butler-Lund et al., 2025). The most frequently reported causes of mortality in previous studies have been reproductive tract infections (e.g. salpingitis-peritonitis, salpingitis, peritonitis, egg (yolk) peritonitis) (Fulton, 2017, 2019; Gretarsson et al., 2023; Butler-Lund et al., 2025), most often due to *E. coli* (Bisgaard and Dam, 1981; Fossum et al., 2009; Stokholm et al., 2010; Butler-Lund et al., 2025), or injurious pecking (e.g. cloacal cannibalism) (Weitzenbürger et al., 2005; Shini et al., 2006; Kajlich et al., 2016; Fulton, 2019; Butler-Lund et al., 2025). Other common causes included emaciation (Shini et al., 2006; Fulton, 2017; Gretarsson et al., 2023; Butler-Lund et al., 2025). However, terminology varied greatly between previous studies, which renders results difficult to compare.

So far, the scarce scientific literature has used two approaches to study causes of mortality in laying hens, 1) reporting tentative causes of death (e.g. Fulton, 2017), or 2) reporting pathological findings without concluding on cause of death (e.g. Kajlich et al., 2016; Zloch et al., 2018) or both (Gretarsson et al., 2023; Butler-Lund et al., 2025). In this study, we aimed to explore associations between pathological findings in dead laying hens with an explorative approach as it may reveal patterns in the data that are not apparent using traditional standard statistical methods. Multiple correspondence analysis provides a data-driven, multidimensional, explorative approach to analyze how pathological findings in laying hens are related and how patterns in combinations of findings in individual hens may create clusters that can be interpreted as diagnoses or tentative causes of death.

The objective of the study was to identify and describe patterns and associations between pathological findings in laying hens that died during the production period using previously obtained data (Butler-Lund et al., 2025). The study also investigated if there are specific pathological findings that farmers can be instructed to look for in dead laying hens to indicate certain causes of mortality and risk factors on bird level. The results could be useful to poultry pathologists and other professionals involved in health management in the layer sector.

## Materials and methods

### Study design, setting and population

Data used in this study was collected in a longitudinal study where we examined 1,801 found dead or euthanized laying hens from seven commercial flocks (three in organic and four in barn housing systems) on six Danish farms in 2020-2022 (Butler-Lund et al., 2025). Characteristics of the seven flocks, including breed, flock size, age at slaughter, cumulative mortality (recorded mortality), egg production and medication in rearing and production period (vaccines, antiparasitics) can be found in Butler-Lund et al. (2025).

As described in Butler-Lund et al. (2025), farmers were instructed to collect the first ten dead hens every other week throughout the production period and store them in freezers (−18 °C) until they could be transported in polystyrene boxes to the University of Copenhagen for necropsy.

Relevant details on necropsies, data recordings and data management are briefly summarized below. For more detailed information on the study design, setting and population, see Butler-Lund et al. (2025).

### Necropsies

As described in Butler-Lund et al. (2025), all *lege artis* necropsies were performed by two experienced poultry pathologists. Prior to necropsy, flock ID and date of death were recorded and each hen was weighed. A total of 59 macroscopic findings in all organ systems were recorded on bird level. These were categorized into 49 categorical variables, see below (Supplementary Table 1). A tentative cause of mortality was recorded based on an overall assessment of the pathological findings and likely pathogeneses in each bird (Supplementary Table 2).

At least one possible tentative cause of mortality was determined for 1,758 hens (97.6%), of which 226 hens (12.9%) could have died from two (*n* = 219), three (*n* = 6) or four (*n* = 1) independent causes. The cause of mortality could not be established for 43 hens (2.4%), either due to no identifiable lesions (*n* = 13) or due to autolysis (*n* = 30). The ten most prevalent tentative causes of mortality were salpingitis-peritonitis (24.8%), (primary) cannibalism (18.3%), egg bound (13.3%), pecking (11.7%), uremia (8.8%), chronic salpingitis (6.3%), septicemia (5.6%), internal hemorrhage (5.3%), gastrointestinal disorders (5.1%), and trauma (3.4%) (Butler-Lund et al., 2025).

#### Data management

As described in Butler-Lund et al. (2025) and Supplementary Table 1, the 59 pathological findings were further categorized into 49 categorical variables to increase the robustness of the results by ensuring at least 20 observations per variable category (Di Franco, 2016) and to avoid non-independence between variables due to data structure. Data editing steps included merging variable categories (16 variables, i.e. plumage, skin lesions, footpad lesions, pericarditis), creating new variables (10 variables, i.e. gastrointestinal tract (GI) tract missing, pale musculature, dark/cyanotic musculature, liver congestion) and consequently recoding variables overlapping with new variables (i.e. the variable categories ‘missing GI tract’ in GI tract related variables), removing variables directly dependent on other variables (3 variables, i.e. age of keel bone fractures and other fractures), removing variables with fewer than 20 observations (8 variables, i.e. persistent right oviduct, liver cirrhosis), or variables with too many missing data (1 variable). Age was categorized in 10-week intervals (≤ 25 weeks, 26-35 weeks, 36-45 weeks, 46-55 weeks, 56-65 weeks, 66-75 weeks and ≥ 76 weeks).

For the MCA to run, we needed to eliminate observations with missing data. Missing data (*n* = 153) were primarily due to hens unfit for necropsy due to autolysis (*n* = 30) or hens not labelled with date of death (*n* = 44). The dataset analyzed with MCA contained 1,648 hens.

#### Data analysis

Multiple correspondence analysis (MCA) is an exploratory multivariate technique designed to study the similarities between individuals, the relationship between variables and variable categories, and to study the link between them in order to characterize the individuals through their pattern of variables (Husson and Josse, 2014). The MCA seeks to represent the cloud of individuals and variables in a low-dimensional Euclidian space by maximizing the variance (inertia). Inertia (a measure of variance) shows the dispersion of data around the center of gravity (Husson and Josse, 2014), in this case the dispersion of individual pathological profiles around the average profile.

An MCA was performed with R, version 4.2.1 (R Core Team 4.2.1, 2022) to summarize and visualize the multidimensional dataset consisting of hens in rows and the pathological findings in columns. The R package FactoMineR was used to perform the analysis and factoextra was used to visualize the results (Lê et al., 2008; Kassambara and Mundt, 2020).

The degree of explained variance provided by each dimension is determined by its eigenvalue. The first dimension will have the highest eigenvalue and gradually decreases with each new dimension. Eigenvalues are used to determine the number of dimensions to include in the interpretation of the MCA, but there is no consensus in the literature on which threshold to use; Greenacre (2007), as cited in Sourial et al. (2010), recommended retaining dimensions with eigenvalues above 1/Q, where Q is the number of variables; in this study, it would mean 27 dimensions explaining 61.79% of the variance. Other authors have suggested not to consider dimensions with eigenvalues below 0.05 (Rodriguez-Sabate et al., 2017), corresponding to retaining the first four dimensions in this study. Due to the low variance explained by each of the remaining dimensions (<3%), we chose to interpret the first four dimensions (Supplementary Figure 1).

The strength of the MCA is that it illustrates graphically the association between individuals, and between variables (and variable categories), the shorter the distance between points, the higher the association. Thus, two variable categories will be close in proximity if individuals with this pathological characteristic are overall more similar on all the variables (Husson and Josse, 2014). When interpreting the most contributing variables and variable categories to the dimension, we chose a cut-off for dimension loadings of 0.3 to interpret relevant variables based on experience from necropsies and biological meaning.

Apart from the (active) variables included in the analysis, it is possible to include supplementary (or illustrative) variables that do not contribute to the formation of the dimensions but are used to ease interpretation of the dimensions. The 49 categorical variables (pathological findings) were included as active variables in the MCA. Seventeen tentative causes of mortality (Supplementary Table 2), housing system, flock ID, euthanasia or not, age category and body weight (in g) were included as supplementary variables in the MCA.

#### Ethical considerations

Farmers’ participation was based on informed consent. Ethics approval was granted by the Animal Ethics Institutional Board (AEIRB) of the Department of Veterinary and Animal Sciences of the University of Copenhagen (ref.no: 2022-12-VCM-019A) and by the Research Ethics Committee for Science and Health of the University of Copenhagen (ref.no: 504-0373/22-5000). The project also complied with the rules of the General Data Protection Regulation, and approval was granted (ref. no.: 514-0724/22-3000).

## Results

### Multiple correspondence analysis

The MCA was performed on a dataset containing 49 pathological findings in 1,648 hens. The first four dimensions were retained, accounting for 20.24% of the variance.

Dimension 1 explained 7.8% of the variance. The most contributing variables (R2 > 0.30, *P* < 0.001) to the formation of the four dimensions are reported in Table 1. Dimensions 2, 3 and 4 explained 4.8%, 4.2% and 3.4% of the variance respectively. Body weight was negatively correlated to Dimension 1 (R2 = −0.21, *P* < 0.001), positively correlated to Dimension 2 (R2 = 0.38, *P* < 0.001) and Dimension 3 (R2 = 0.46, *P* < 0.001).

**Table 1:** Variables with dimension loadings (R2) > 0.30 for Dimensions (Dim) 1-4 of the multiple correspondence analysis and pathological findings (variable categories) and pathologists’ diagnoses of the tentative cause of mortality in 20 highest and 20 lowest scoring hens on each dimension of 1,648 laying hens that died during the production period.

Dim	Variable	R2	Variable categories and pathologists’ diagnoses in 20 lowest scoring hens (frequency)	Variable categories and pathologists’ diagnoses in 20 highest scoring hens (frequency)
1	Oviduct	0.57	Missing oviduct (20)	Salpingitis (15)
				Congestion (3)
				Chronic salpingitis (2)
	Nephropathy	0.49	Normal kidneys (15)	Severe nephropathy (20)
			Moderate nephropathy (4)	
			Severe nephropathy (1)	
	Peritonitis	0.45	Normal peritoneum (20)	Fibrinopurulent (17)
				Adhesive (and fibrinopurulent) (3)
	Dark/cyanotic musculature	0.42	No (20)	Yes (19)
				No (1)
	In lay	0.40	In lay (17)^a^	Partial ovarian regression (10)
			Active ovary, no egg in oviduct (3)	Total ovarian regression (7)
				Active ovary, no egg in oviduct (3)
	Pale musculature	0.39	Yes (19)	No (20)
			No (1)	
	Ovary	0.39	Normal (16)	Oophoritis (19)
			Ovary missing (3)	Normal (1)
			Congestion (1)	
	Dehydration	0.38	No (19)	Yes (20)
			Yes (1)	
	Cause of mortality		Cannibalism (19)	Salpingitis-peritonitis (14)
			Cannibalism and trauma (1)	Acute salpingitis-peritonitis (1)
				Chronic salpingitis-peritonitis (1)
				Salpingitis-peritonitis and polyserositis (2)
				Chronic salpingitis (1)
				Chronic salpingitis and polyserositis (1)
2	Oviduct	0.71	Juvenile oviduct (19)	Salpingitis (12)
			Regressive oviduct (1)	Missing oviduct (5)
				Congestion (3)
	In lay	0.42	Juvenile ovary (18)	Partial ovarian regression (10)
			Total ovarian regression (2)	In lay (5)^a^
				Active ovary, no egg in oviduct (4)
				Total ovarian regression (1)
	Body condition	0.35	Emaciated (18)	Obese (9)
			Below normal (2)	Normal (7)
				Above normal (3)
				Below normal (1)
	Cause of mortality		Gastrointestinal disorders (11)	Salpingitis-peritonitis (11)
			Uremia (3)	Cannibalism (4)
			No diagnoses (3)	Salpingitis-peritonitis and polyserositis (2)
			Bumble foot and/or arthritis (2)	Salpingitis-peritonitis and cannibalism (2)
			Anemia (1)	Acute salpingitis-peritonitis (1)
3	Oviduct	0.67	Missing oviduct (19)	Congestion (19)
			Total ovarian regression (1)	Salpingitis (1)
	Gastrointestinal tract missing	0.40	Yes (20)	No (20)
	Cause of mortality		Cannibalism (13)	Egg bound (11)
			Cannibalism and uremia (4)	Salpingitis-peritonitis (3)
			Cannibalism and salpingitis-peritonitis (2)	Salpingitis-peritonitis and polyserositis and internal hemorrhage (1)
			Cannibalism and trauma (1)	Salpingitis-peritonitis and internal hemorrhage
				
				Acute salpingitis-peritonitis (1)
				Polyserositis (1)
				Gastrointestinal disorders (1)
				Cardiovascular disorders (1)
4	Spleen congestion	0.36	No (20)	Yes (18)
				No (2)
	Endocarditis	0.34	No (20)	Yes (19)
				No (ventricular dilatation) (1)
	Liver congestion	0.32	No (20)	Yes (19)
				No (1)
	Cause of mortality		Salpingitis-peritonitis (16)	Sepsis (19)
			Chronic salpingitis (3)	Salpingitis-peritonitis (1)
			Acute salpingitis-peritonitis (1)	

a Hens with missing ovary were recoded as in lay to avoid multiple recordings of the same lesion in the variables In lay and Ovary.

The degree of association between variable categories and the dimension axes is illustrated in Supplementary Figure 2 (A-B) as the quality of representation (squared cosine, cos2) of the variable categories, for Dimensions 1 and 2, and 3 and 4, respectively. These variance plots illustrate the complexity of pathological findings in laying hens and suggest how causes of mortality relate to pathological findings.

**Fig. 1:**
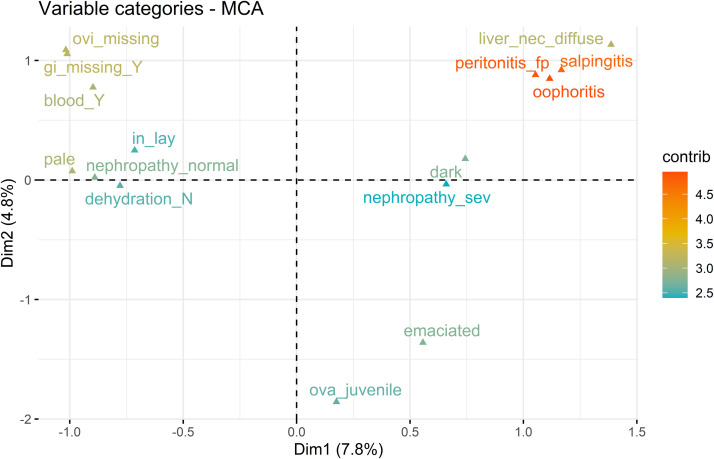
Plot of the correlation between the 15 most contributing variable categories of 49 pathological findings (active variables, labels colored according to their contribution (contrib)) and Dimensions 1 (Dim1) and 2 (Dim2) of the multiple correspondence analysis of pathological findings in 1,648 Danish laying hens that died during the production period. The categories characterizing hens at the positive pole of Dimension 1 were: oophoritis, fibrinopurulent peritonitis (peritonitis_fp), salpingitis, diffuse liver necrosis (liver_nec_diffuse), dark/cyanotic musculature (dark), severe nephropathy (nephropathy_sev) and emaciated body condition (emaciated). The categories characterizing hens at the negative pole of Dimension 1 were: oviduct missing (ovi_missing), gastrointestinal (GI) tract missing (GI_missing_Y), pale musculature (pale), blood at the cloacal region (blood_Y), no nephropathy (nephropathy_normal), no dehydration (dehydration_N) and in lay (in_lay). The categories characterizing hens at the positive pole of Dimension 2 were: diffuse liver necrosis (liver_nec_diffuse), oviduct missing (ovi_missing), gastrointestinal (GI) tract missing (GI_missing_Y), oophoritis, fibrinopurulent peritonitis (peritonitis_fp), salpingitis and blood at the cloacal region (blood_Y). The categories characterizing hens at the negative pole of Dimension 2 were: juvenile ovary (ova_juvenile) and emaciated body condition (emaciated).

**Fig. 2:**
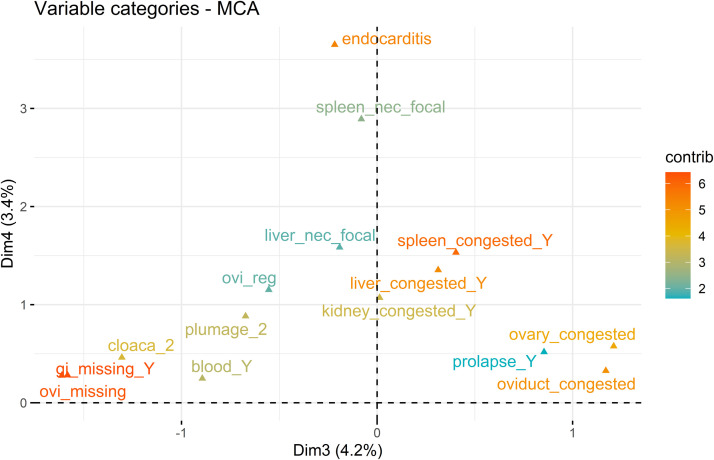
Plot of the correlation between the 15 most contributing variable categories of 49 pathological findings (active variables, labels colored according to their contribution (contrib)) and Dimensions 3 (Dim3) and 4 (Dim4) of the multiple correspondence analysis of pathological findings in 1,648 Danish laying hens that died during the production period. The categories characterizing hens at the positive pole of Dimension 3 were: congestion of the ovary (ovary_congested), congestion of the oviduct (oviduct_congested) and cloacal prolapse (prolapse_Y). The categories characterizing hens at the negative pole of Dimension 3 were: oviduct missing (ovi_missing), gastrointestinal (GI) tract missing (GI_missing_Y), cloacal lesions with necrosis (cloaca_2), blood at the cloacal region (blood_Y) and more than 25% of visible skin (plumage_2). The categories characterizing hens at the positive pole of Dimension 4 were: endocarditis, focal spleen necrosis (spleen_nec_focal), focal liver necrosis (liver_nec_focal), congestion of the spleen (spleen_congested_Y), congestion of the liver (liver_congested_Y), total regression of the oviduct (ovi_reg), congestion of the kidney (kidney_congested_Y), more than 25% of visible skin (plumage_2) and ovary (ovary_congested). None of the 15 most contributing variable categories characterized the negative pole of Dimension 4.

**Fig. 3:**
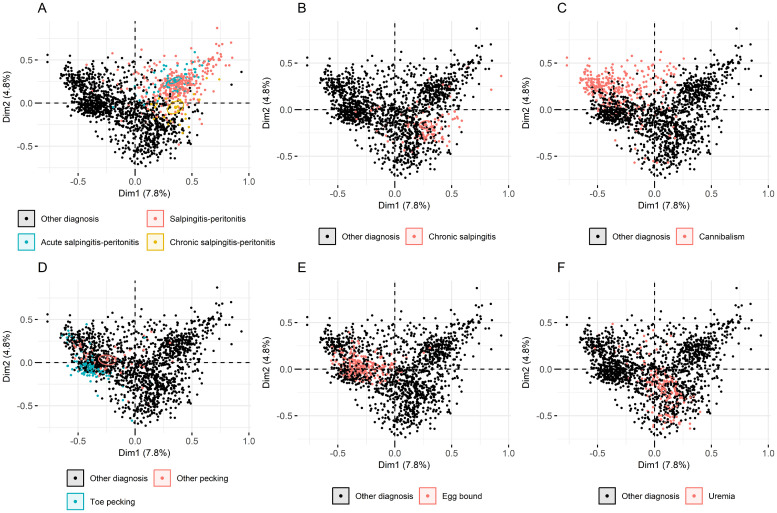
Plot of the correlation between hens and Dimensions 1 (Dim1) and 2 (Dim2) of the multiple correspondence analysis of 49 pathological findings in 1,648 Danish laying hens that died during the production period. The distribution of six causes of mortality (supplementary variables) are represented: (A) salpingitis-peritonitis (salpingitis-peritonitis, acute salpingitis-peritonitis, chronic salpingitis-peritonitis) or other diagnosis; (B) chronic salpingitis or other diagnosis; (C) cannibalism or other diagnosis; (D) pecking (toe pecking, other types of pecking (other pecking)) or other diagnosis; (E) egg bound or other diagnosis; (F) uremia or other diagnosis. The central tendency (barycenter) and its 95% confidence ellipse is represented for each cause of mortality. The correlation between the dimension axes and the variable categories are considered significantly different when the confidence ellipses are not overlapping (Husson et al., 2017).

**Fig. 4:**
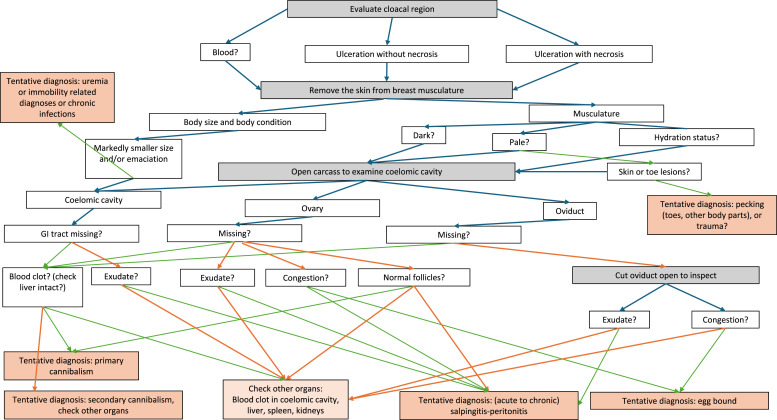
An example of a simplified guide to necropsy of found dead or euthanized laying hens in field situations. Green arrows symbolize the next step after a positive finding and red arrows after a negative finding.

To visualize the dimensions, we plotted the 15 most contributing variable categories to the formation of Dimension 1 and 2 (Fig. 1), and 3 and 4 (Fig. 2) respectively. Fig. 1 shows that the positive pole of Dimension 1 is associated with the following pathological findings (variables categories): oophoritis, fibrinopurulent peritonitis, salpingitis, and to a lesser extent, diffuse liver necrosis, dark/cyanotic musculature and severe nephropathy. Oophoritis, fibrinopurulent peritonitis and salpingitis are cardinal signs in hens diagnosed with salpingitis-peritonitis as a cause of mortality (Supplementary Table 2). The negative pole of Dimension 1 was associated with missing organs (gastrointestinal and/or reproductive tract), blood in the cloacal region, pale musculature and, to a lesser extent, normal kidneys, active ovary and normal hydration status. To conclude, Dimension 1 described a spectrum of pathological findings distinguishing hens with infectious disease (e.g. cardinal signs of salpingitis-peritonitis) from hens with non-infectious disorders (e.g. injurious pecking, including signs of cloacal cannibalism (evisceration and hemorrhaging). Whereas Dimension 2 described chronicity represented by body condition (juvenile and emaciated) at the negative pole and in lay (active ovary) at the positive pole.

Fig. 2 shows that the positive pole of Dimension 3 was associated with: congestion in oviduct and ovary and, to a lesser extent, cloacal prolapse, which are cardinal signs of egg bound (Supplementary Table 2). The negative pole was associated with missing organs (gastrointestinal and/or reproductive tract), cloacal lesions with necrosis and, to some extent, blood in the cloacal region and poor plumage, which may also be considered cardinal signs of cannibalism (Supplementary Table 2). Finally, the positive pole of Dimension 4 was associated with: endocarditis, congestion in the spleen, liver, kidneys and, to a lesser extent, focal necrosis in the spleen and liver and ovarian regression, which are cardinal signs of septicemia (Supplementary Table 2). None of the 15 most contributing variable categories contributed significantly to the negative pole of Dimension 4.

### Association between dimensions and diagnoses by pathologists

The association between hens diagnosed with salpingitis-peritonitis (A) and/or chronic salpingitis (B), cannibalism (C) and/or pecking (D) and/or egg bound (E) and/or uremia (F), as a cause of mortality and Dimension 1 and 2 is illustrated in Fig. 3. Confirming the interpretation of Fig. 1, Fig. 2, Dimension 1 captured, on the negative pole, non-infectious disorders (injurious pecking (including cannibalism and pecking at toes or other body parts (Fig. 3C-D)), egg bound (Fig. 3E), trauma (data not shown) and internal hemorrhage (data not shown). These may be considered conditions with a primarily acute to subacute course (Butler-Lund et al., 2025). The positive pole of Dimension 1 captured infectious disorders (Fig. 3A-B). The pathological findings of hens diagnosed with salpingitis-peritonitis and acute salpingitis-peritonitis did not differ, whereas they clearly differed from hens diagnosed with chronic salpingitis-peritonitis, which were plotted in proximity to hens diagnosed with chronic salpingitis (Fig. 3B) and to some extent uremia (Fig. 3F) (without an apparent primary lesion). The ellipses mark the central tendency (barycenter) with 95% confidence of individual hens according to their coordinates on the axes of Dimensions 1 and 2.

The association between diagnoses and Dimensions 3 and 4 were also plotted and showed clustering corresponding to the diagnoses, but less clearly defined than for Dimensions 1 and 2 (data not shown).

To further characterize the dimensions, we extracted the scores on the main contributing variables and variable categories and the ten most common diagnoses of the 20 highest and 20 lowest scoring hens in each of the four dimensions, as reported in Table 1. The variable categories of the highest and lowest scoring individuals confirmed the interpretation of Figs. 1 and 2, while the frequencies of the diagnoses supported the interpretation of Fig. 3.

Based on the above, Dimensions 1 to 4 were interpreted as follows:

Dimension 1: **Infectious / non-infectious**: Hens scoring high presented with pathological findings suggesting infectious disorders (localized to the reproductive tract with sequel systemic signs, e.g. acute-chronic salpingitis-peritonitis), whereas hens scoring low presented with pathological findings suggesting non-infectious disorders (primarily related to injurious pecking (e.g. cannibalism)).

Dimension 2: **Acute / Chronic**: Hens scoring high were characterized by being in lay (and associated with higher body weight) (acute to subacute course, e.g. salpingitis-peritonitis or cannibalism), whereas hens scoring low were characterized by emaciated body condition (chronic courses, e.g. gastrointestinal disorders, immobility).

Dimension 3: **Reproductive tract / cannibalism**: Hens scoring high presented with oviductal congestion (and higher body weight) suggesting reproductive tract disorders (e.g. egg bound, salpingitis-peritonitis), whereas hens scoring low presented with missing reproductive and gastrointestinal tract (e.g. cannibalism, some with other pathologies).

Dimension 4: **Systemic / localized infectious:** Hens scoring high presented with congestion of parenchymatous organs and endocarditis (e.g. septicemia), whereas hens scoring low presented with localized infections of the reproductive tract.

### Characteristics of clusters of hens diagnosed with cannibalism

Dimensions 1-3 suggested at least two clusters of pathological findings in hens diagnosed with cannibalism. Hens with the lowest scores on Dimension 1 rarely had other diagnoses than cannibalism (cannibalism as the single cause of mortality), had pale musculature suggesting hemorrhaging and no signs of concurrent pathologies. Hens with the highest scores on Dimension 2 in some cases had concurrent salpingitis and signs of beginning regression of the ovary (cannibalism with signs of concurrent acute-subacute pathologies). Hens with the lowest scores on Dimension 3 in some cases had concurrent pathologies of a more chronic nature, including deteriorated body condition (cannibalism with signs of concurrent chronic pathologies).

To further characterize the different clusters of hens diagnosed with cannibalism, we plotted the individual hens on Dimension 1 and 2 labelled by variables (and variable categories) related to cannibalism (Supplementary Figure 3 A-H). This suggested two clusters of hens diagnosed with cannibalism in the upper left and into the upper right quadrant: 1) acute cases characterized by normal kidneys and cloacal lesions without necrosis, primarily intact plumage, blood in the cloacal region, pale musculature, above normal body condition and active ovary, and 2) prolonged cases characterized by moderate to severe nephropathy, cloacal lesions with necrosis and less frequently intact plumage, blood in the cloacal region and pale musculature, and varying body condition and a higher frequency of hens with partial ovarian regression (Supplementary Figure 3 A-H). Similarly, in Dimension 3 and 4, two clusters were also visible, where the more prolonged cases were scoring highest on the negative axis of Dimension 3, as opposed to on Dimension 1 (data not shown). The two clusters observed in Dimension 1 and 2 differed slightly from the two clusters observed in Dimension 3 and 4 in terms of association to body condition, hen age and ovarian status. It may be partly related to flock differences (e.g. body weight).

The acute cannibalism cluster did not show a clear age-dependency, whereas the more prolonged cluster tended to include older age groups of hens (data not shown).

### Association between dimensions and housing system, hen age and euthanasia

Dimensions 1 and 2 were associated with hen age. Hens aged > 66 weeks tended to be located in the upper right quadrant (acute to subacute infectious disease), whereas the lower left quadrant (egg bound and pecking) was related to hens aged < 35 weeks (data not shown).

Dimensions 3 and 4 were also associated with hen age. Hens aged > 66 weeks tended to be located in the upper left quadrant (primarily prolonged cases of cannibalism). Whereas hens aged < 35 weeks were associated with the positive pole of Dimension 3 (similarly to hens diagnosed with egg bound) (data not shown). Flock variation was apparent as hens from flock A scored higher than others on Dimension 4.

Euthanasia was associated with the negative pole of Dimension 2 (similarly to hens diagnosed with uremia) (data not shown).

### Association between exterior pathology and mortality causes in laying hens

We also ran an MCA with only the externally assessable findings (e.g. plumage, skin and cloacal lesions, toe and footpad lesions, cleanliness of the cloacal region) (data not shown). One dimension was retained for interpretation (accounting for 10.4% of the variance). The most contributing variables (R2 > 0.30) were cloacal lesions (0.63) and blood at the cloacal region (0.59).

That only about 10% of the variance could be explained by exterior pathology, may suggest high variability between observations and low association with causes of mortality.

## Discussion

This study contributes with new insights into pathological findings and diagnostic criteria in laying hens that died during the production period. By using an exploratory multivariate approach to analyzing pathological data, we suggest (at least) two distinct types of cannibalism based on association between pathological findings in dead laying hens. Unlike descriptive statistics, the MCA illustrates the complexity of pathological processes, while identifying major clusters of hens with similar pathological findings corresponding to the most prevalent diagnoses previously reported in these hens (Butler-Lund et al., 2025).

### Added value of multivariate analysis

Compared to the descriptive analysis reported in Butler-Lund et al. (2025), the MCA supported the main results by clustering hens with diagnoses of similar expected chronicity and general etiology (infectious versus non-infectious). This may be unsurprising as the diagnoses were based on necropsy findings in the same hens, why the pathologists’ diagnoses (supplementary variables) were merely used to interpret the results of the MCA, not to validate them. However, the most dominant clusters identified corresponded to the two most common causes of mortality identified in previous studies, namely salpingitis-peritonitis, commonly due to *E. coli*, and cannibalism (Stokholm et al., 2010; Fulton, 2017, 2019; Gretarsson et al., 2023; Butler-Lund et al., 2025). The diagnoses were extensively described and discussed by Butler-Lund et al. (2025). However, the MCA added nuance to the variability of pathological findings in hens to improve the understanding of mortality causes, e.g., the two clusters of cannibalism were not visible in the descriptive analysis. These may point to relevant differences in etiology and pathogenesis that should be explored in future studies.

Contrary to this study, most previous studies reported one diagnosis per hen (Stokholm et al., 2010; Fulton, 2017, 2019), potentially simplifying the pathological manifestations. While a simplified approach may ease focus on specific diagnoses, taking all pathological manifestations into account provided us with the opportunity to investigate how pathological findings cluster, irrespective of predefined diagnoses. Previous studies infrequently reported underlying pathological findings, which hampers comparability of results.

The transparency of this reporting helped identify multiple clusters of hens with signs of cannibalism. This finding may inspire future research into etiology and pathogenesis of cannibalism in laying hens. In addition, our results also supported the finding of Butler-Lund et al. (2025), that hens diagnosed with cannibalism frequently have intact plumage. The MCA suggested that intact plumage was mostly related to the acute cluster of cannibalized hens.

However, some pathognomonic signs of prevalent diagnoses were not captured by the MCA (i.e. toe pecking lesions in hens diagnosed with toe pecking as the cause of mortality), which could be due to high variability between these hens in other findings and/or subtle pathological findings (ventricular dilatation, pallor) not specifically enough described by our variables.

A prominent finding in Butler-Lund et al. (2025) was the association of egg bound and above normal body condition. That the MCA did not suggest a high loading of body condition for dimensions (1 and 3) describing signs of egg bound (oviductal and ovarian congestion) may be due to the recorded variable categories not being specific to egg bound hens (oviduct congestion also observed in hens with acute salpingitis), that egg bound was frequently observed concurrently with other diagnoses (22.5% (Butler-Lund et al., 2025)), or that pathologists may also have interpreted other signs, than those recorded, to diagnose the condition. However, body weight was positively correlated with Dimension 3, supporting that higher-than-normal body condition is associated with egg bound hens. Body weight of laying hens increases over the production period (e.g. Lohmann, 2025), and the MCA suggested that individuals clustering in proximity to egg bound hens, were also clustering close to hens of younger age groups, thus supporting the interpretation that positive correlation with body weight in egg bound hens also suggests a correlation with higher-than-normal body condition. Little is known about the etiology and pathogenesis of egg binding in laying hens (Butler-Lund et al., 2025) and we suggest that further research is warranted.

Finally, the MCA suggested that the recorded pathological findings did not differ between hens diagnosed with acute and subacute salpingitis-peritonitis, and that hens diagnosed with chronic salpingitis-peritonitis and chronic salpingitis had similar pathological profiles. This may mean that these represent different stages of the same pathological process, adding to the discussion of diagnostic criteria and variability of pathological manifestations in hens suffering from the likely most common cause of mortality (Olsen et al., 2016).

### Guidance for field necropsies of laying hens

That external findings had a relatively low loading on the four dimensions accounting for most of the variance in the dataset, may indicate that external findings are not suitable to distinguish between specific causes of mortality in laying hens (except for blood at the cloacal region indicating cannibalism). Cloacal lesions (R2 = 0.29) had relatively high loading on Dimension 3 and was the highest loading variable in the MCA on external findings only. We suggest that examination of the cloacal region of dead hens (i.e. blood, ulcers with or without necrosis) could be a first step when screening for cannibalism in dead hens. However, necropsy is needed to distinguish between primary and secondary cannibalism (if the hen was alive during the cannibalistic attack). Pale musculature was not consistently observed amongst hens in the cluster with prolonged course of cannibalism which may be due to other pathological processes masking pallor (i.e., cyanosis due to uremia). This may also be an example of why the MCA was able to explain relatively little of the variance in the dataset.

That external characteristics recorded in this study were not highly correlated to causes of mortality may also suggest that clinical signs in hens are subtle and may be difficult to use for farmers to assess the need for euthanasia of individuals. Euthanasia was correlated with the negative pole of Dimension 2, similarly to hens diagnosed with uremia (with unknown cause) and chronic infections, which suggests that farmers are mostly euthanizing hens with chronic pathologies (and emaciated body condition).

The results of this study may be useful to identify pathological findings to focus on, in field necropsies where time-efficiency is essential to screen dead hens for the most common diagnoses to guide further investigations and/or to monitor the effectiveness of preventative and control measures. Fig. 4 presents a flow diagram of the highest loading variables and the most likely diagnoses based on the MCA to guide field necropsies. Our findings suggest that evaluation of the reproductive organs and coelomic cavity is of particular importance for diagnostics. These examinations may be performed by veterinarians, but farmers could possibly be trained to recognize pathological signs for initial self-monitoring. Continuous systematic monitoring of causes of mortality, e.g. (primary) cannibalism, in combination with specialist sparring (e.g. veterinarians, nutritionists etc.) may also benefit awareness and prompt farmers to consider preventative measures. Recent qualitative research indicated that layer hen farmers underestimate the occurrence of cannibalism (Butler-Lund et al., unpublished data).

Finally, the results suggested an age-dependency in pathological findings (and consequently diagnoses). We observed two associations for the oldest age groups of hens: Dimensions 1-2 suggested acute to subacute infectious disease related to the reproductive tract, while Dimensions 3-4 suggested cannibalism of a prolonged course. On the other hand, the youngest age groups were associated with egg bound and pecking.

If these age dependencies could be interpreted as risk periods for certain mortality causes needs further research as flock variation, seasonality and housing system may confound the results.

### Acute or prolonged course of cannibalism

That cannibalism had a relatively poor association with pathological findings other than missing gastrointestinal and reproductive tract may suggest a certain variability in pathological findings in these birds. Butler-Lund et al. (2025) found that 28.6% of hens diagnosed with cannibalism also showed pathological manifestations compatible with other causes of mortality. Our results suggest that these hens were part of the cluster with a prolonged course of cannibalism. It may also be that some relevant findings were not systematically recorded in our study (e.g., bloody feathers, comb color).

None of the sampled flocks (based on dead hens) had very poor plumage and individuals with 75% or more visible skin were rare (*n* = 14, 0.8%). Our results suggested that, in particular, the acute cluster of cannibalism was not associated with plumage and that many cannibalized hens had intact plumage. As pointed out by Butler-Lund et al. (2025) and others before (Pötzsch et al., 2001; Lambton et al., 2015), the etiology and pathogenesis of cannibalism constitute important knowledge gaps with implications for prevention and control of mortality and impaired welfare in laying hens. Our results indicated that at least two different variants of cannibalism may exist. We consider these results highly relevant in terms of providing direction for future research into etiology and pathogenesis of cannibalism in laying hens.

Some previous studies have suggested an association between salpingitis and cannibalism in laying hens (Cumming, 2001; Pötzsch et al., 2001). Our results only partially supported this theory, as Dimension 1 placed hens diagnosed with cannibalism and salpingitis on opposite poles, except for some overlap on the positive pole of Dimension 2 (prolonged cases of cannibalism primarily), possibly suggesting that salpingitis may predispose to cannibalism, though causality could not be established in our study design. A complication to evaluating the association between cannibalism and salpingitis is that the oviduct is often missing in cannibalized hens. Other findings may guide the diagnosis in these cases (e.g. oophoritis, nephropathy, muscular discoloration).

### Recommendations for control and prevention of most common causes of mortality

**Fig. 5:**
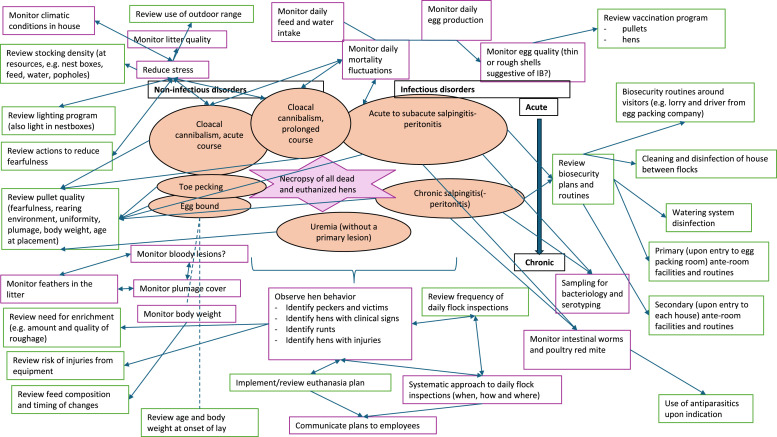
Recommendation for a flock health monitoring plan for laying hens. We recommend systematic necropsy of dead hens to assess mortality causes (which are likely to include the pathologies marked with orange fill and arranged similarly to clusters suggested by the MCA (Dimensions 1 and 2)) and thereby target control and preventative measures to current health and behavioral problems. Actions marked by purple outlines are recommended to be continuously undertaken by farmers, while actions with green outlines are recommended if prompted by necropsy results and to be undertaken/discussed in collaboration with advisors. Actions are derived from recent (yet unpublished) research (Butler-Lund et al. (2025), unpublished data) and other previous studies (Pötzsch et al., 2001; Linares et al., 2018; Cronin and Glatz, 2020; Edwards and Hemsworth, 2021; Vasdal et al., 2022; Mels et al., 2022, 2023; Schreiter et al., 2022; Butler-Lund et al., 2025).

Fig. 5 presents some recommendations based on our recent research and previous studies that may aid farmers and their advisors (veterinarians and others) to decide on possible preventative and control measures that may be relevant to combat the most prevalent diagnoses according to the results of this study. We recommend using the approach suggested in Fig. 4 on as many dead hens as possible to continuously monitor the currently most prevalent causes of mortality in the flock throughout the production period. Based on results of this study and Butler-Lund et al. (2025), we expect that the majority of hens would fall into at least one of the tentative diagnosis categories or clusters suggested in Fig. 5. However, several knowledge gaps still exist regarding control and prevention of the most common causes of mortality, e.g., etiology and pathogenesis of (potentially two types of) cannibalism and egg bound. Recent research has suggested a health monitoring scheme for pullets and we suggest that many of the principles can be extended to laying hens (Mels et al., 2022, 2023), such as monitoring of feathers in the litter as an indicator of feather eating and feather pecking. We suggest that an increased focus on quantity and quality of daily flock inspections, including monitoring hen behavior, may increase the likelihood of early detection of health and behavioral problems, e.g. identify peckers and victims, runts etc. to be euthanized. Systematic flock inspection methods may aid in increasing the quality of flock inspections (Vasdal et al., 2022). We suggest that the recommendations in Fig. 5 could be discussed and evaluated together with a veterinarian to create, implement, or evaluate a flock health plan for each new laying hen flock placed.

### Bias and study limitations

MCA offers a time efficient multivariate analysis methodology compared to univariate analyses, such as regression models. As we wanted to study patterns in pathological findings without a predefined outcome, a multivariate approach was needed.

That the MCA explained relatively little of the variance in the data, suggested that pathological findings and their correlations are complex and that clustering is limited, as also exemplified by the relatively low dimension loadings of the highest loading variables (many R2 < 0.40) (Table 1). That some hens had multiple diagnoses (e.g. cannibalism (28.6%) (Butler-Lund et al., 2025)) may have limited clustering that could be due to individual diagnoses. Salpingitis-peritonitis was one of the diagnoses most often present without other diagnoses (13.0% (Butler-Lund et al., 2025)), which may have enhanced clustering. Nephropathy was both a sequel to other diagnoses and a diagnostic criterion of uremia, which may have limited clustering. By including hens with multiple diagnoses, we were able to suggest associations between causes of death that may suggest new directions for research into etiologies and pathogeneses, while also allowing the results to reflect actual pathological findings in individual birds (where comorbidities may exist) and thereby illustrate the complexity of pathological findings where some pathological processes may mask others (e.g. pallor due to hemorrhage may be masked by cyanosis due to uremia). None-the-less, the MCA identified clusters of related pathological findings, and the suggested clusters corresponded to the most prevalent diagnoses of pathologists on the same hens, as presented in a previous study (Butler-Lund et al., 2025). The results reflect choices made before necropsies, on which variables to record and at what level of detail, i.e., we did not distinguish between different exudates in the oviduct. Furthermore, the merging and recoding of variables to increase the number of observations per category might have affected clustering. However, data editing also aimed to limit non-independence between variables caused by structure of the recorded data rather than pathological processes. Different variable construction schemes were tested to assess their impact on the MCA results. However, as the variance explained by the first dimensions of the MCA did not change substantially before and after data editing, it seemed to have little relevant impact on the results. Finally, the results are expected to reflect biological variation between hens.

According to other studies, there are no clear guidelines on the minimum number of cases required to perform an MCA (Husson and Josse, 2014; Di Franco, 2016). We ensured a minimum of 20 observations in each variable category, as recommended by Di Franco (2016). However, the ratio of variable categories to observations in our study may have contributed to the limited clustering observed (Di Franco, 2016).

The MCA mimicked the complex process of the pathologist to determine the cause of mortality based on the pathological findings observed during *lege artis* necropsies. Two experienced pathologists performed all necropsies of hens for this study. Although the variables and variable categories were chosen to ensure consistency and repeatability in the recording of pathological findings, inter- and intra-rater agreement may not be perfect. We did not record the identity of the pathologist as part of this study, why inter- and intra-rater agreement could not be explored. The lengthy study period (> 2 years) may have been a disadvantage in terms of intra-rater agreement but was prerequisite to study mortality over the full production period. There is a lack of reporting guidelines for poultry pathology in literature. Such guidelines may also increase comparability of results of future scientific studies.

## Conclusion

To conclude, this study offered a rarely used, yet useful, approach to investigate pattern recognition in, and associations between, pathological findings in laying hens that died during the production period. Despite some methodological limitations, the MCA results suggested clusters of infectious and non-infectious disorders of differing chronicity, pointing to the most commonly reported causes of mortality in laying hens in the scarce scientific literature, namely salpingitis-peritonitis and cannibalism. Adding to the insights obtained by descriptive analysis, the MCA results indicated at least two clusters of hens with similar pathological findings suggesting either an acute or a prolonged course leading to cannibalism. This highlights a need to investigate etiology and pathogenesis of cannibalism in laying hens further, also to validate the existence of such distinct types of cannibalism. We suggested guidance to performing time-efficient field necropsies to screen for the most common causes of mortality to improve monitoring of these conditions in commercial flocks. Improved monitoring is a prerequisite for targeted control and preventative measures, and we suggested recommendations for farmers and their advisors (veterinarians and others) to target control and prevention to the most prevalent causes of mortality in laying hens.

## CRediT authorship contribution statement

**Vibe P. Butler-Lund:** Writing – review & editing, Writing – original draft, Visualization, Validation, Resources, Project administration, Methodology, Investigation, Formal analysis, Data curation, Conceptualization. **Liza R. Nielsen:** Writing – review & editing, Validation, Supervision, Methodology, Conceptualization. **Ida C.N. Thøfner:** Writing – review & editing, Supervision, Investigation, Conceptualization. **Jens P. Christensen:** Writing – review & editing, Supervision, Project administration, Investigation, Funding acquisition, Conceptualization.

## Disclosures

The authors declare that they have no known competing financial interests or personal relationships that could have appeared to influence the work reported in this paper.

## Appendix Supplementary materials

Image, application 1 dummy alt text

Image, application 2 dummy alt text

Image, application 3 dummy alt text

Image, application 4 dummy alt text

Image, application 5 dummy alt text

## Footnotes

Supplementary material associated with this article can be found, in the online version, at doi:10.1016/j.psj.2026.106734.
