# Supplementary material for: A multiple correspondence analysis of necropsy findings in non-caged laying hens that died during the production period
Source: Poult Sci. 2026 Mar 3;105(6):106734. doi: 10.1016/j.psj.2026.106734 (PMC13067112; doi:10.1016/j.psj.2026.106734)

Supplementary Figure 1. Scree plot showing the percentage of explained variance per dimension for the first 10 dimensions (of 73 dimensions) of the multiple correspondence analysis of 49 pathological findings in 1,648 Danish laying hens that died during the production period.

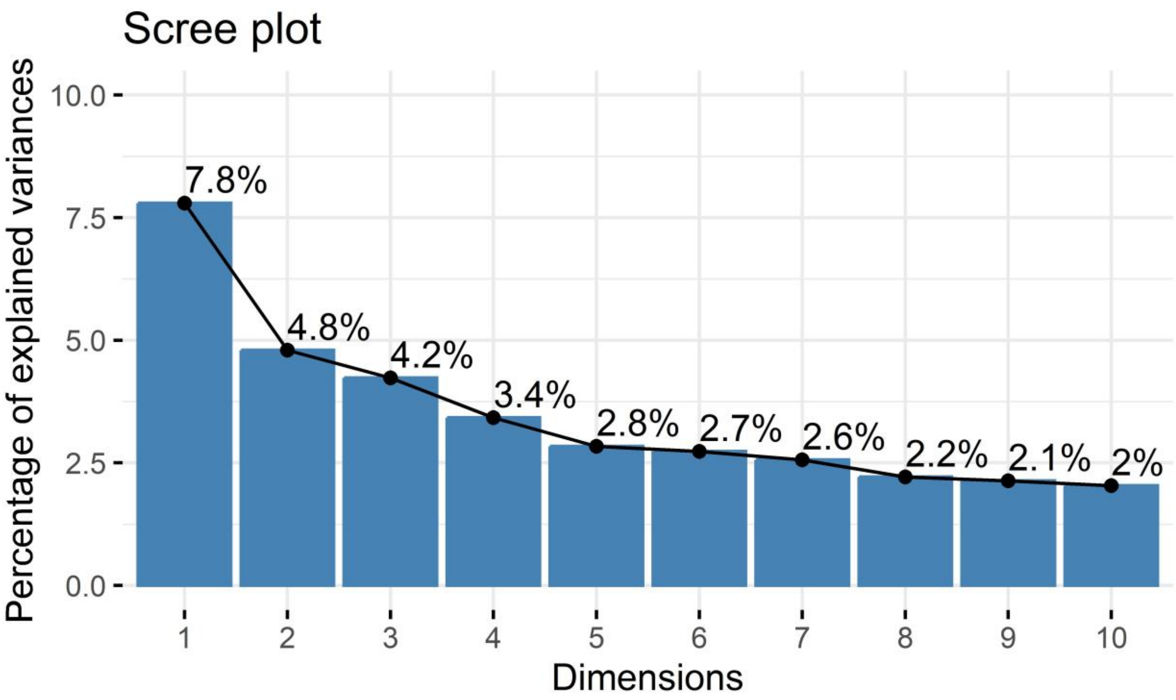

Supplement: Supplementary file 1 [file mmc1.pdf]
