# Supplementary figures and images for: A multiple correspondence analysis of necropsy findings in non-caged laying hens that died during the production period
Source: Poult Sci. 2026 Mar 3;105(6):106734. doi: 10.1016/j.psj.2026.106734 (PMC13067112; doi:10.1016/j.psj.2026.106734)

# B

### Variable categories - MCA

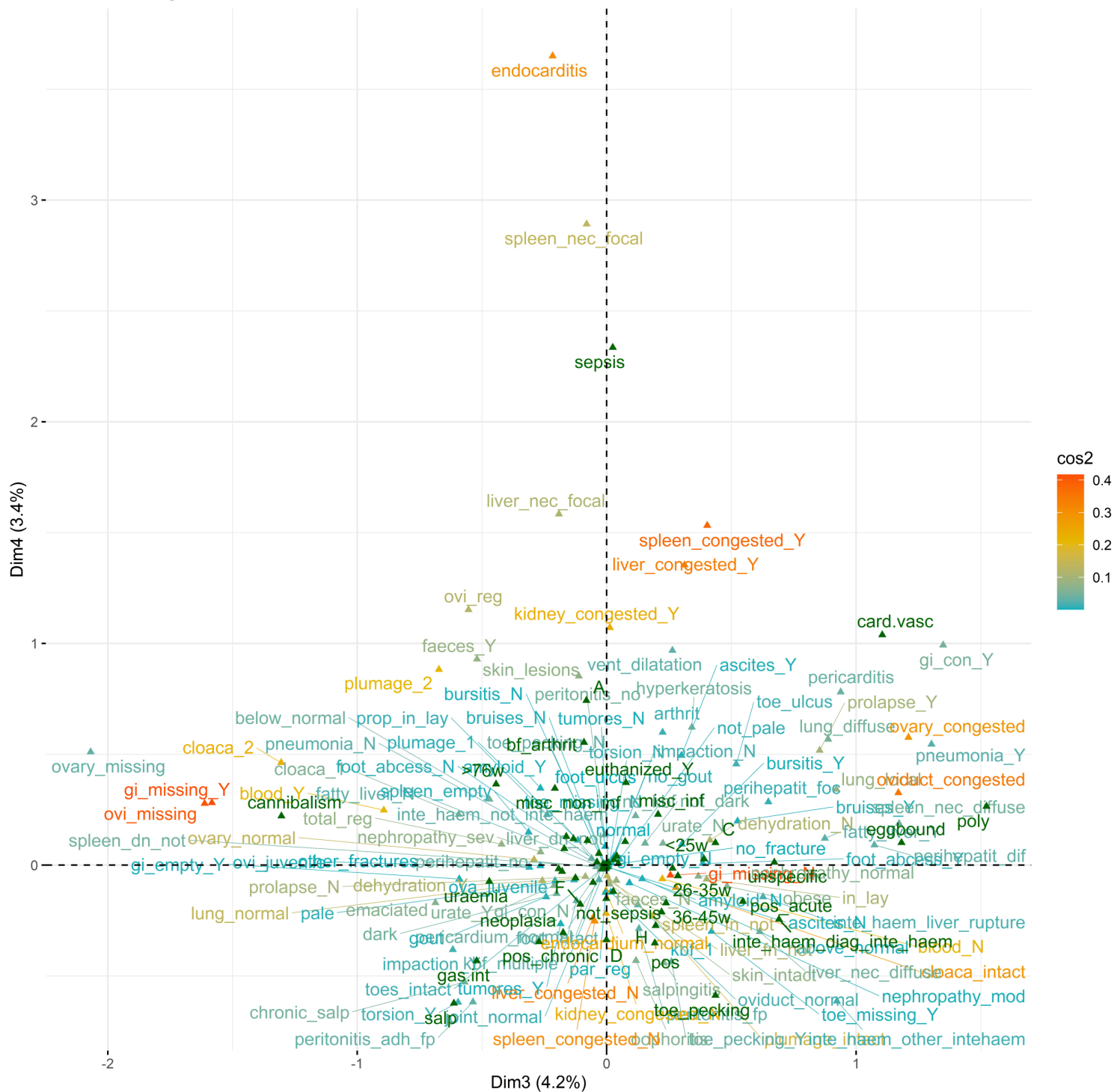

Supplement: Supplementary file 2 [file mmc2.pdf]
