# Supplementary material for: A multiple correspondence analysis of necropsy findings in non-caged laying hens that died during the production period
Source: Poult Sci. 2026 Mar 3;105(6):106734. doi: 10.1016/j.psj.2026.106734 (PMC13067112; doi:10.1016/j.psj.2026.106734)
Supplement: Supplementary file 3 [file mmc3.pdf]

Supplementary Figure 3. Plot of the correlation between hens and Dimensions 1 (Dim1) and 2 (Dim2) of the multiple correspondence analysis of 49 pathological findings in 1,648 Danish laying hens that died during the production period. The distribution of hens diagnosed with cannibalism (or any other diagnosis) (A) and seven pathological findings are represented: (B) plumage (intact, < 25% visible skin or > 25% visible skin), (C) blood in cloacal region (yes, no), (D) cloacal lesion (no, yes, without necrosis or yes, with necrosis), (E) pale musculature (yes, no), (F) nephropathy (none, moderate, severe), (G) body condition (emaciated, below normal, normal, above normal, obese), (H) in lay (yes (active ovary, egg in oviduct), likely (active ovary, no egg in oviduct), partial ovarian regression, total ovarian regression, juvenile). The central tendency (barycenter) and its 95% confidence ellipse are represented for each pathological finding. The correlation between the dimension axes and the variable categories is considered significantly different when the confidence ellipses are not overlapping (Husson et al., 2017).

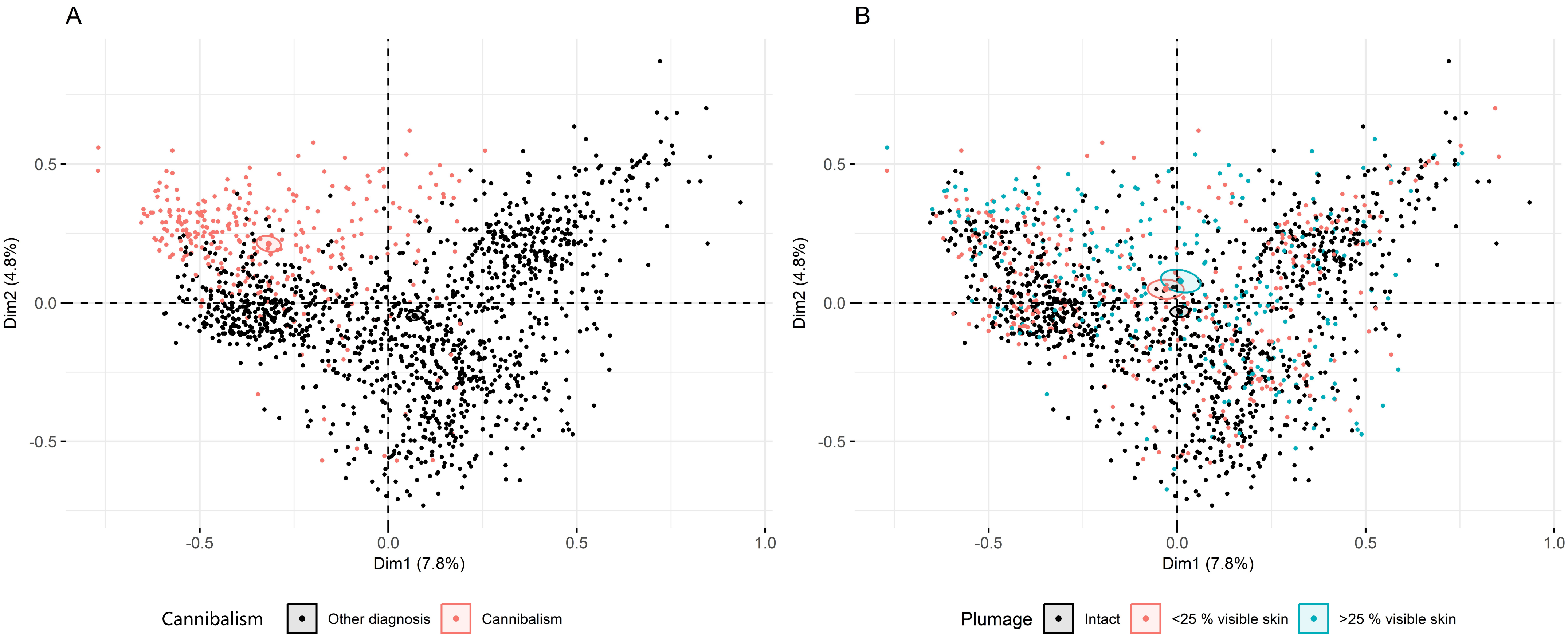

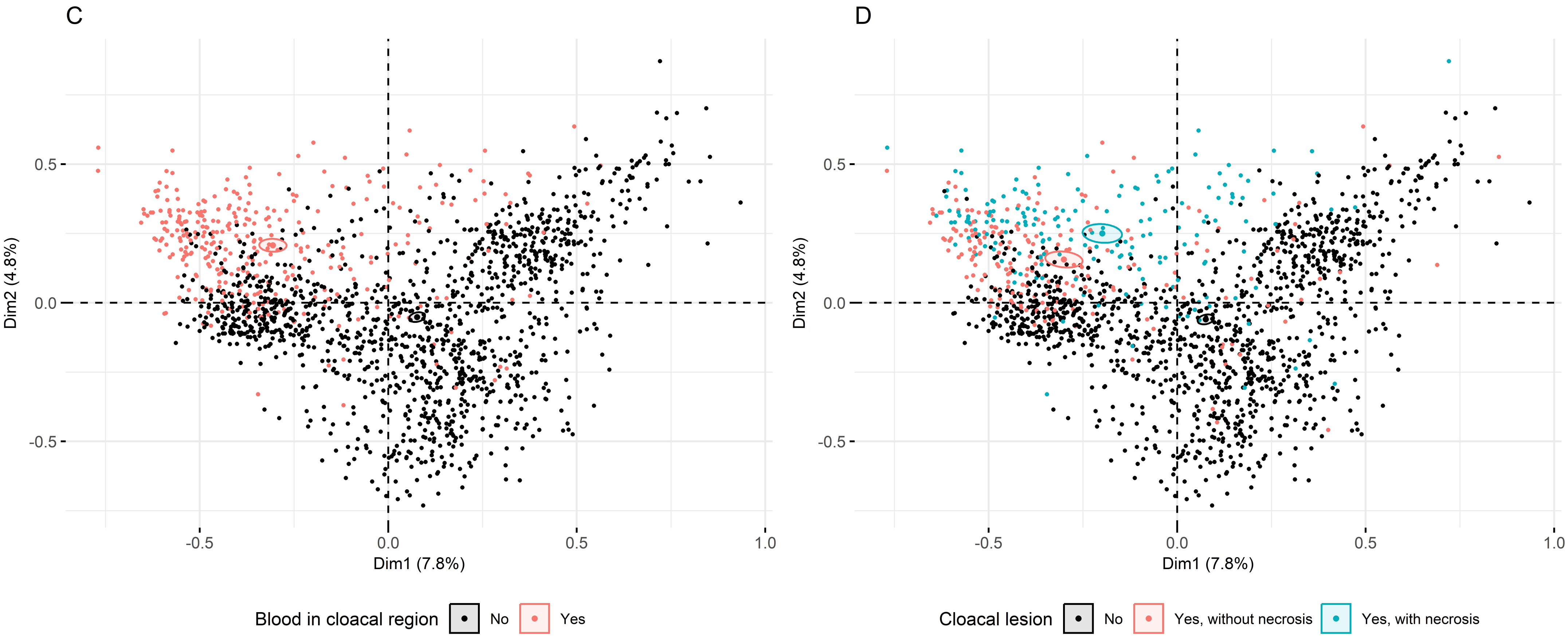
C
Dim2 (4.8%)
Dim1 (7.8%)
0.5
0.0
-0.5
-0.5
0.0
0.5
1.0
Blood in cloacal region
No
Yes
D
Dim2 (4.8%)
Dim1 (7.8%)
0.5
0.0
-0.5
-0.5
0.0
0.5
1.0
Cloacal lesion
No
Yes, without necrosis
Yes, with necrosis

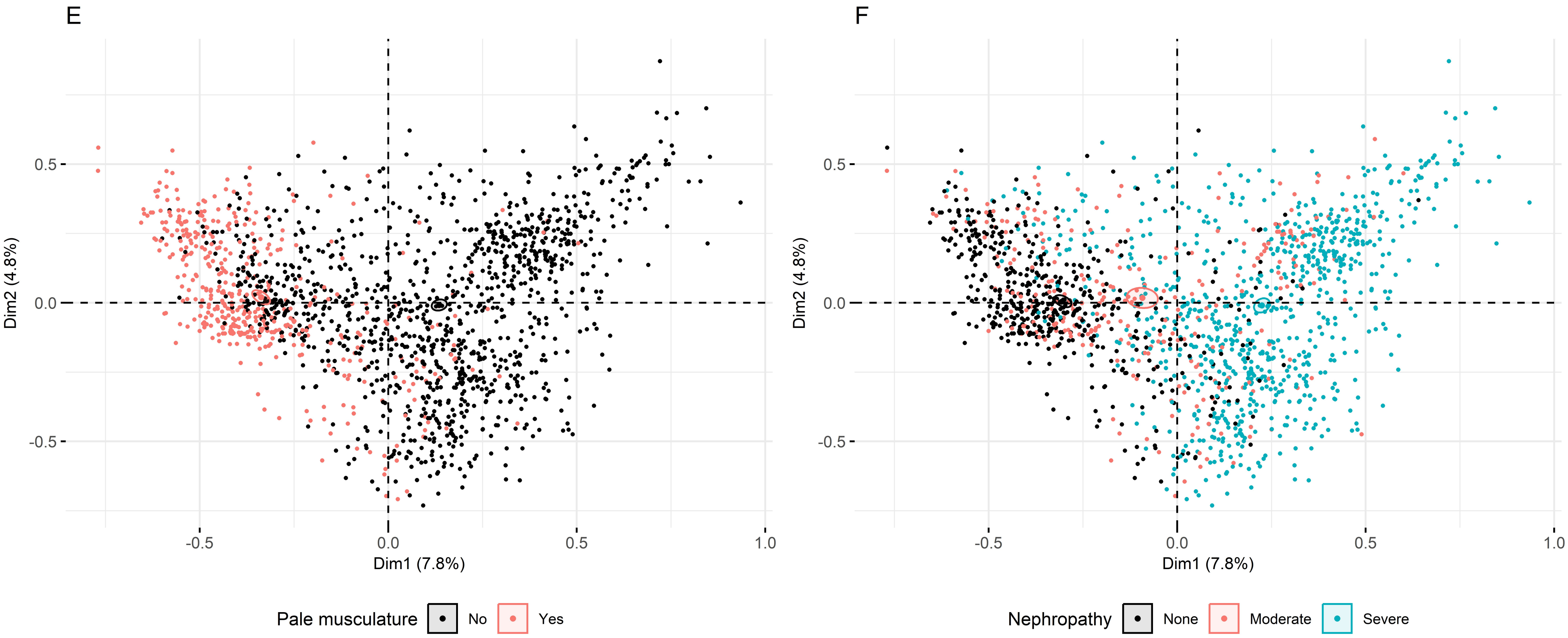
E
F
Dim1 (7.8%)
Dim2 (4.8%)
-0.5
0.0
0.5
1.0
Pale musculature
No
Yes
Nephropathy
None
Moderate
Severe

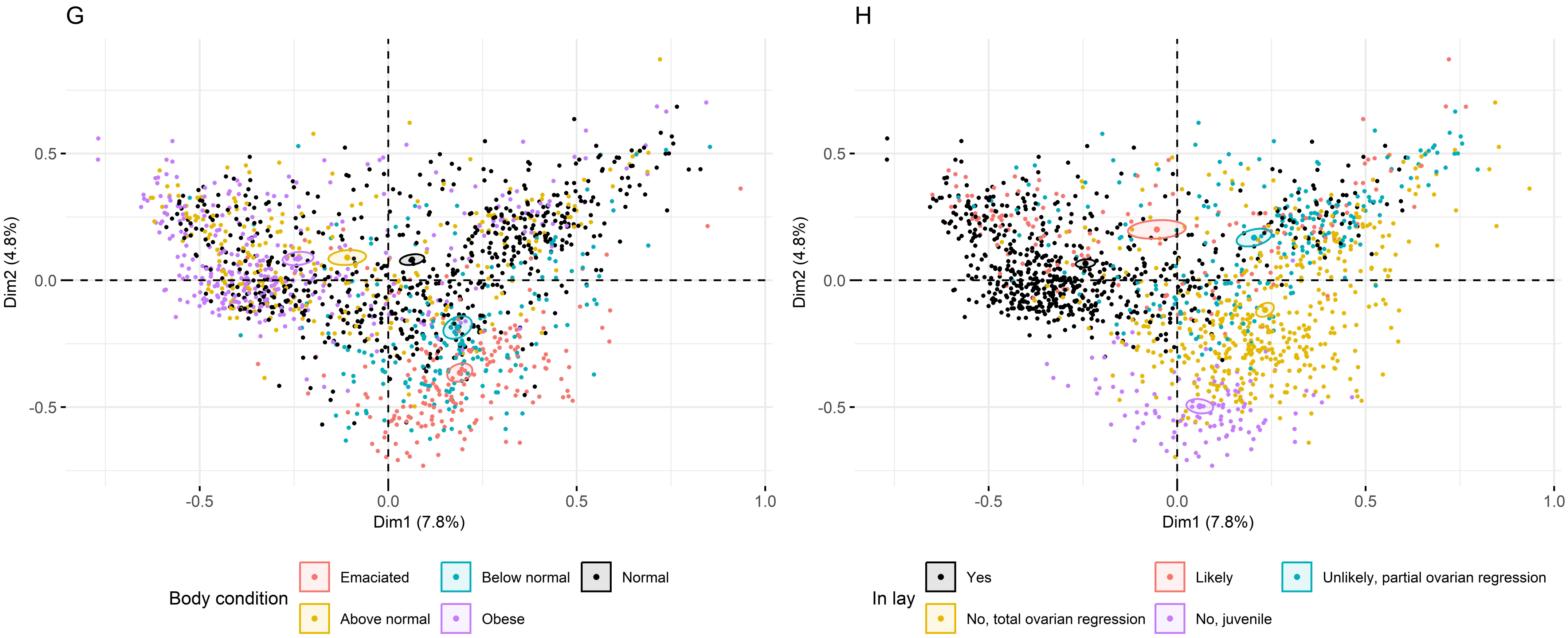
G
H
Dim1 (7.8%)
Dim2 (4.8%)
-0.5
0.0
0.5
1.0
Body condition
Emaciated
Below normal
Normal
Above normal
Obese
In lay
Yes
Likely
Unlikely, partial ovarian regression
No, total ovarian regression
No, juvenile
