# Supplementary material for: A multiple correspondence analysis of necropsy findings in non-caged laying hens that died during the production period
Source: Poult Sci. 2026 Mar 3;105(6):106734. doi: 10.1016/j.psj.2026.106734 (PMC13067112; doi:10.1016/j.psj.2026.106734)
Supplement: Supplementary file 4 [file mmc4.pdf]

Supplementary Table 1. Pathological findings in 1,801 Danish laying hens that died during production. Recorded data (59 variables) are displayed in the left columns and aggregated data (49 variables) in the rights columns (modified from Butler-Lund et al., (2025))

**Recorded data (n=1801)**					**Aggregated data (n=1801)**			
**Variable**	**Scale**	**Number of hens**	**% of all hens necropsied**		**Variable**	**Scale**	**Number of hens**	**% of all hens necropsied**
**Plumage**[a]	0: fully feathered	1156	64.2		**Plumage**	0: fully feathered	1156	64.2
	1: <25 % visible skin	362	20.1			1: <25 % visible skin	362	20.1
	2: 25-75% visible skin	250	13.9			2: =>25% visible skin	264	14.7
	3: >75% visible skin)	14	0.8			Not recorded	19	1.1
	Not recorded	19	1.1					
**Skin lesions**[b]	0: none	1620	90.0		**Skin lesions**	0: none	1620	90.0
	1: small single lesion (=<2 cm diam)	57	3.2			1: one or more skin lesions	162	9.0
	2: large (>2 cm diam) or multiple smaller	105	5.8			Not recorded	19	1.1
	Not recorded	19	1.1					
**Urate**	0: no	865	48.0		**Urate**	0: no	865	48.0
	1: yes	918	51.0			1: yes	918	51.0
	Not recorded	18	1.0			Not recorded	18	1.0
**Feces**	0: no	1648	91.5		**Feces**	0: no	1648	91.5
	1: yes	135	7.5			1: yes	135	7.5
	Not recorded	18	1.0			Not recorded	18	1.0
**Blood**	0: no	1419	78.8		**Blood**	0: no	1419	78.8
	1: yes	364	20.2			1: yes	364	20.2
	Not recorded	18	1.0			Not recorded	18	1.0
**Bile**	0: no	1778	98.7					
	1: yes	5	0.3					
	Not recorded	18	1.0					

**Peritoneal fluid**	0: no	1777	98.7					
	1: yes	6	0.3					
	Not recorded	18	1.0					
**Cloacal prolapse**	0: no	1619	89.9		**Cloacal prolapse**	0: no	1619	89.9
	1: yes	164	9.1			1: yes	164	9.1
	Not recorded	18	1.0			Not recorded	18	1.0
**Cloacal lesions**[c]	0: none	1354	75.2		**Cloacal lesions**	0: none	1354	75.2
	1: yes, without necrosis	228	12.7			1: yes, without necrosis	228	12.7
	2: yes, with necrosis	201	11.2			2: yes, with necrosis	201	11.2
	Not recorded	18	1.0			Not recorded	18	1.0
**Footpad lesions**[d]	0: none	1494	83.0		**Footpad lesions**	0: none	1494	83.0
	1: hyperkeratosis	137	7.6			1: hyperkeratosis	137	7.6
	2: ulcer/necrosis <=2mm	33	1.8			2: ulcer/necrosis	153	8.5
	3: ulcer/necrosis >2mm	120	6.7			Not recorded	17	0.9
	Not recorded	17	0.9					
**Bumble foot**	0: no	1725	95.8		**Foot abscess**	0: none	1715	95.2
	1: yes	59	3.3			1: footpad or toe abscess	69	3.8
	Not recorded	17	0.9			Not recorded	17	0.9
**Toe lesions (not considered due to toe pecking)**	0: no	1719	95.4		**Toe lesions (not considered due to toe pecking)**	0: no	1732	96.2
	1: ulceration	52	2.9			1: ulcer/necrosis	52	2.9
	2: abscessation	13	0.7			Not recorded	17	0.9
	Not recorded	17	0.9					
**Toe pecking lesion**	0: no	1576	87.5		**Toe pecking lesion**	0: no	1576	87.5

	1: yes	208	11.5			1: yes	208	11.5
	Not recorded	17	0.9			Not recorded	17	0.9
**Toe missing**	0: no	1755	97.4		**Toe missing**	0: no	1755	97.4
	1: yes, one or more claws or toes	30	1.7			1: yes, one or more claws or toes	30	1.7
	Not recorded	16	0.9			Not recorded	16	0.9
**Joint lesions**	0: no	1724	95.7		**Joint lesions**	0: no	1724	95.7
	1: arthritis, single joint	39	2.2			1: arthritis, one or multiple joints	50	2.8
	2: arthritis, multiple joints	11	0.6			Not recorded	27	1.5
	Not recorded	27	1.5					
**Body condition**[e]	0: normal	682	37.9		**Body condition**[e]	0: normal	682	37.9
	1: below normal	210	11.7			1: below normal	210	11.7
	2: emaciated	207	11.5			2: emaciated	207	11.5
	3: above normal	300	16.7			3: above normal	300	16.7
	4: obese	375	20.8			4: obese	375	20.8
	Not recorded	27	1.5			Not recorded	27	1.5
**Dehydration**	0: no	697	38.7		**Dehydration**	0: no	697	38.7
	1: yes	1073	59.6			1: yes	1073	59.6
	Not recorded	31	1.7			Not recorded	31	1.7
**Discoloration of musculature**	0: none	503	27.9		**Pale musculature**	0: no	1249	69.4
	1: pale	512	28.4			1: yes	520	28.9
	2: dark/cyanosis	746	41.4			Not recorded	32	1.8
	3: pale and dark/cyanosis	8	0.4		**Dark/cyanotic musculature**	0: no	1015	56.4
	Not recorded	32	1.8			1: yes	754	41.9
						Not recorded	32	1.8
**Bursitis presternalis**	0: no	1718	95.4		**Bursitis presternalis**	0: no	1718	95.4

	1: yes	52	2.9			1: yes	52	2.9
	Not recorded	31	1.7			Not recorded	31	1.7
**Keel bone fractures**[f]	0: no fractures	696	38.6		**Keel bone fractures**	0: no fractures	696	38.6
	1: one fracture	334	18.5			1: one fracture	334	18.5
	2: two fractures	294	16.3			2: multiple fractures	745	41.4
	3: three fractures	267	14.8			Not recorded	26	1.4
	4: four fractures	102	5.7					
	5+: five or more fractures	82	4.6					
	Not recorded	26	1.4					
**Age of keel bone fractures**[g]	0: no callus	693	38.5					
	1: fresh fracture w/wo bleeding	34	1.9					
	2: minimal callus	302	16.8					
	3: moderate callus	739	41.0					
	Not recorded	33	1.8					
**Ossification of keel bone**	0: fully ossified	1572	87.3					
	1: <= 1cm cartilage	128	7.1					
	2: > 1cm cartilage	72	4.0					
	Not recorded	29	1.6					
**Bruises**	0: no	1745	96.9		**Bruises**	0: no	1745	96.9
	1: yes	26	1.4			1: yes	26	1.4
	Not recorded	30	1.7			Not recorded	30	1.7
**Other fractures**	0: none	1726	95.8		**Other fractures**	0: none	1726	95.8
	1: pelvis	9	0.5			1: one or multiple fractures	45	2.5
	2: ribs	3	0.2			Not recorded	30	1.7
	3: leg	23	1.3					

	4: wing	3	0.2					
	5: keel (external)	2	0.1					
	6: multiple	5	0.3					
	Not recorded	30	1.7					
**Age of other fracture**	0: none	1655	91.9					
	1: fresh fracture w/wo bleeding	22	1.2					
	2: minimal callus	4	0.2					
	3: moderate callus	10	0.6					
	Not recorded	110	6.1					
**Peritonitis**	0: none	1265	70.2		**Peritonitis**	0: none	1265	70.2
	1: exudative (fibrinopurulent, purulent and/or hemorrhagic)	417	23.2			1: exudative (fibrinopurulent, purulent and/or hemorrhagic)	417	23.2
	2: adhesive	70	3.9			2: adhesive (and exudative)	79	4.4
	3: exudative + adhesive	9	0.5			Not recorded	40	2.2
	Not recorded	40	2.2					
**Intra-abdominal hemorrhage**[h]	0: no	1516	84.2		**Intra-abdominal hemorrhage**	0: no	1516	84.2
	1: liver rupture	97	5.4			1: liver rupture	97	5.4
	2: ovarian rupture	20	1.1			2: ovarian or kidney rupture	42	2.3
	3: hemorrhage around kidneys	22	1.2			3: other origin	106	5.9
	4: other origin	106	5.9			Not recorded	40	2.2
	Not recorded	40	2.2					
**Lung congestion**	0: no	1548	86.0		**Lung congestion**	0: no	1548	86.0
	1: localized (<25%)	138	7.7			1: localized (<25%)	138	7.7
	2: diffuse	73	4.1			2: diffuse	73	4.1

	Not recorded	42	2.3			Not recorded	42	2.3
**Pneumonia/airsacculitis**	0: no	1734	96.3		**Pneumonia/airsacculitis**	0: no	1734	96.3
	1: fibrinopurulent	24	1.3			1: yes	24	1.3
	Not recorded	43	2.4			Not recorded	43	2.4
**Pericarditis**	0: no	1718	95.4		**Pericarditis**	0: no	1718	95.4
	1: focal fibrinous pericardium	12	0.7			1: yes	41	2.3
	2: diffuse fibrinous pericardium	25	1.4			Not recorded	42	2.3
	3: purulent/fibrinopurulent pericardium	4	0.2					
	Not recorded	42	2.3					
**Lesions of the endocardium**	0: none	1665	92.4		**Endocarditis**	0: none	1671	92.8
	1: ventricular dilatation (left/right)	50	2.8			1: ventricular dilatation (left/right)	50	2.8
	2: endocarditis (mural or valvular)[i]	38	2.1			2: endocarditis (mural or valvular)	38	2.1
	3: necrosis in myocardium	5	0.3			Not recorded	42	2.3
	4: neoplasia	1	0.1					
	Not recorded	42	2.3					
**Ascites**	0: no	1737	96.4		**Ascites**	0: no	1737	96.4
	1: yes	21	1.2			1: yes	21	1.2
	Not recorded	43	2.4			Not recorded	43	2.4
**Torsion, intussusception etc.**	0: no	1476	82.0		**Torsion, intussusception etc.**	0: no	1728	95.9
	1: yes	31	1.7			1: yes	31	1.7

	2: GI tract missing	252	14.0			Not recorded	42	2.3
	Not recorded	42	2.3					
**Impaction (mechanical) of proventriculus, gizzard, duodenum, jejunum, ileum**	0: no	1455	80.8		**Impaction (mechanical) of proventriculus, gizzard, duodenum, jejunum, ileum**	0: no	1707	94.8
	1: yes	52	2.9			1: yes	52	2.9
	2: GI tract missing	252	14.0			Not recorded	42	2.3
	Not recorded	42	2.3					
**Crop impaction or pendulous crop**	0: no	1490	82.7		**Empty intestines**	0: no	1737	96.4
	1: yes	17	0.9			1: yes	22	1.2
	2: GI tract missing	252	14.0			Not recorded	42	2.3
	Not recorded	42	2.3					
**Empty intestines**	0: no	1485	82.5		**Congestion (in intestinal wall)**	0: no	1729	96.0
	1: yes	22	1.2			1: yes	30	1.7
	2: GI tract missing	252	14.0			Not recorded	42	2.3
	Not recorded	42	2.3					
**Congestion (in intestinal wall)**	0: no	1477	82.0		**GI tract missing**	0: no	1507	83.7
	1: yes	30	1.7			1: yes	252	14.0
	2: GI tract missing	252	14.0			Not recorded	42	2.3
	Not recorded	42	2.3					
**Enteritis**	0: no	1503	83.5					
	1: yes	4	0.2					
	2: GI tract missing	252	14.0					

	Not recorded	42	2.3					
**Neoplasia**	0: no	1743	96.8					
	1: yes	16	0.9					
	Not recorded	42	2.3					
**In lay**	0: yes (presence of egg/yolk in oviduct and fully active ovary)	686	38.1		**In lay**	0: yes (presence of egg/yolk in oviduct and fully active ovary)	705	39.1
	1: likely (no egg/yolk in oviduct, but fully active ovary)	163	9.1			1: likely (no egg/yolk in oviduct, but fully active ovary)	163	9.1
	2: unlikely (no egg/yolk in oviduct, 1 or more regressive follicles)	247	13.7			2: unlikely (no egg/yolk in oviduct, 1 or more regressive follicles)	247	13.7
	3: no, total ovarian regression	523	29.0			3: no, total ovarian regression	523	29.0
	4: no, juvenile	120	6.7			4: no, juvenile	120	6.7
	5: ovary fully or partly missing	19	1.1			Not recorded	43	2.4
	Not recorded	43	2.4					
**Oviduct**[j]	0: no lesion	487	27.0		**Oviduct**	0: no lesion	494	27.4
	1: congestion, edema, discoloration	331	18.4			1: congestion, edema, discoloration	331	18.4
	2: exudative salpingitis (fibrinopurulent, purulent and/or hemorrhagic), with or without congestion	336	18.7			2: exudative salpingitis (fibrinopurulent, purulent and/or hemorrhagic), with or without congestion	336	18.7
	3: distended (large accumulation of inflammatory exudate), chronic salpingitis	136	7.6			3: distended (large accumulation of inflammatory exudate), chronic salpingitis	136	7.6
	4: juvenile	76	4.2			4: juvenile	76	4.2
	5: regressive	121	6.7			5: regressive	121	6.7

	6: fully or partly missing	241	13.4			6: fully or partly missing	241	13.4
	7: neoplasia	7	0.4			Not recorded	66	3.7
	Not recorded	66	3.7					
**Persistent right oviduct**	0: no	1757	97.6					
	1: yes (cystic, chronic distended salpingitis etc.)	15	0.8					
	Not recorded	29	1.6					
**Ovary**	0: no	1087	60.4		**Ovary**	0: no	1098	61.0
	1: generalized congestion	236	13.1			1: generalized congestion	236	13.1
	2: exudative (fibrinopurulent, purulent and/or hemorrhagic) oophoritis, with or without congestion	403	22.4			2: exudative (fibrinopurulent, purulent and/or hemorrhagic) oophoritis, with or without congestion	403	22.4
	3: fully or partly missing	19	1.1			3: fully or partly missing	19	1.1
	4: neoplasia	11	0.6			Not recorded	45	2.5
	Not recorded	45	2.5					
**Perihepatitis**	0: no	1679	93.2		**Perihepatitis**	0: no	1679	93.2
	1: focal, fibrinous	24	1.3			1: focal	32	1.8
	2: focal, fibrinopurulent	8	0.4			2: diffuse	45	2.5
	3: diffuse, fibrinous	38	2.1			Not recorded	45	2.5
	4: diffuse, fibrinopurulent	7	0.4					
	Not recorded	45	2.5					
**Hepatomegaly**	0: no	1434	79.6		**Liver congestion**	0: no	1501	83.3
	1: yes, congestion	256	14.2			1: yes	256	14.2
	2: yes, amyloid	41	2.3			Not recorded	44	2.4

	3: neoplasia	26	1.4					
	Not recorded	44	2.4					
**Liver necrosis**	0: no	1535	85.2		**Liver necrosis, focal**	0: none	1685	93.6
	1: focal	67	3.7			1: focal	71	3.9
	2: diffuse	150	8.3			Not recorded	45	2.5
	3: focal and diffuse	4	0.2		**Liver necrosis, diffuse**	0: none	1602	89.0
	Not recorded	45	2.5			1: diffuse	154	8.6
**Liver cirrhosis**	0: no	1753	97.3			Not recorded	45	2.5
	1: yes	4	0.2					
	Not recorded	44	2.4					
**Fatty liver**	0: no	1695	94.1		**Fatty liver**	0: no	1695	94.1
	1: yes	60	3.3			1: yes	60	3.3
	Not recorded	46	2.6			Not recorded	46	2.6
**Splenomegaly**	0: no	1411	78.3		**Spleen congestion**	0: no	1457	80.9
	1: yes, congestion	221	12.3			1: yes	221	12.3
	2: yes, amyloid	28	1.6			3: empty, pale, small spleen	80	4.4
	3: empty, pale, small spleen	80	4.4			Not recorded	43	2.4
	4: neoplasia	18	1.0					
	Not recorded	43	2.4					
**Spleen necrosis**	0: no	1680	93.3		**Spleen necrosis, focal**	0: none	1731	96.1
	1: focal	26	1.4			1: focal	27	1.5
	2: diffuse	51	2.8			Not recorded	43	2.4
	3: focal and diffuse	1	0.1		**Spleen necrosis, diffuse**	0: none	1706	94.7
	Not recorded	43	2.4			1: diffuse	52	2.9

						Not recorded	43	2.4
**Renomegaly**	0: no	1397	77.6		**Kidney congestion**	0: no	1455	80.8
	1: yes, congestion	285	15.8			1: yes	285	15.8
	2: yes, amyloid	36	2.0			Not recorded	61	3.4
	3: neoplasia	22	1.2					
	Not recorded	61	3.4					
**Nephropathy**	0: none	554	30.8		**Nephropathy**	0: none	578	32.1
	1: moderate (increased tubular pattern or swelling or urate in ureter or pale)	300	16.7			1: moderate (increased tubular pattern or swelling or urate in ureter or pale)	300	16.7
	2: severe (at least two of the following: increased tubular pattern, swelling, urate in ureter, pale)	873	48.5			2: severe (at least two of the following: increased tubular pattern, swelling, urate in ureter, pale)	873	48.5
	3: atresia/hypoplasia (partial) of one kidney AND severe nephropathy of other kidney	15	0.8			Not recorded	50	2.8
	4: missing (due to cannibalism)	9	0.5					
	Not recorded	50	2.8					
**Visceral gout**	0: no	1646	91.4		**Visceral gout**	0: no	1646	91.4
	1: some	21	1.2			1: yes	108	6.0
	2: manifest/severe	87	4.8			Not recorded	47	2.6
	Not recorded	47	2.6					
**Amyloid**	0: no	1716	95.3		**Amyloid**	0: no	1716	95.3
	1: articular	4	0.2			1: yes	44	2.4
	2: systemic incl articular	37	2.1			Not recorded	41	2.3
	Not recorded	41	2.3					

**Generalized circulatory disturbances**	0: no	1626	90.3					
	1: yes	93	5.2					
	Not recorded	82	4.6					
**Neoplasia**[k]	0: no	1709	94.9		**Neoplasia**	0: no	1709	94.9
	1: yes	62	3.4			1: yes	62	3.4
	Not recorded	30	1.7			Not recorded	30	1.7
	**Minimum**	**1st quantile**	**Median**	**Mean**	**3rd quantile**	**Maximum**	**Not recorded**	
**Body weight (in g)**	412	1274	1454	1434	1622	2355	2	

[a] Scale modified from Tauson et al., (2005).

[b] Scale modified from Hinrichsen et al., (2016).

[c] Lesions included ulcerations, lacerations, hyperemia and/or oedema.

[d] Scale modified from Hinrichsen et al., (2016).

[e] Scale modified from Gregory & Robins, (1998) and Grafl et al., (2017). Normal: round breast muscle, limited keel protuberance; Below normal: relatively well-developed breast muscle, distinct keel protuberance; Emaciated: prominent keel bone ridge, scarce breast muscle; Above normal: very round breast musculature, no keel protuberance; Obese: obese, internal fat in abdominal cavity.

[f] Scale modified from Thøfner et al., (2021).

[g] Scale modified from Thøfner et al., (2021). If multiple keel bone fractures of different ages (n=17), the oldest was recorded.

[h] If liver rupture was observed together with hemorrhage of ovarian or kidney origin (n=3), liver rupture was recorded.

[i] In four cases, hens with endocarditis also showed signs of myocardial necrosis. These were recorded as endocarditis.

[j] If salpingitis or chronic salpingitis occurred in hens with a regressive (n=5) or juvenile (n=1) oviduct, salpingitis or chronic salpingitis were recorded respectively.

[k] Diffuse or focal (solitary or multiple) cellular infiltrations in one or more organs: liver, spleen, kidneys, ovary, oviduct, mesentery, pancreas, Bursa Fabricii etc. Neoplasia was also recorded at organ level (e.g. as a category in the variable ‘Splenomegaly’), but due to too few observations at organ level and to avoid non-independence, only the dichotomous ‘Neoplasia’ variable was retained for analysis. Consequently, the categories of neoplasia at organ level were recoded to the no lesion/no category (e.g. ‘Oviduct’) or not included in the newly created variables (e.g. ‘Spleen congestion’). The former seemed plausible as these organs otherwise appeared normal and to avoid non-independence due to multiple recordings of the same lesions.
