# Supplementary material for: A multiple correspondence analysis of necropsy findings in non-caged laying hens that died during the production period
Source: Poult Sci. 2026 Mar 3;105(6):106734. doi: 10.1016/j.psj.2026.106734 (PMC13067112; doi:10.1016/j.psj.2026.106734)
Supplement: Supplementary file 5 [file mmc5.pdf]

Supplementary Table 2. Definitions of tentative causes of mortality in 1801 Danish laying hens that died during production in 2020-2022 (modified from Butler-Lund et al., (2025))

Infectious causes of mortality	Salpingitis-peritonitis	Exudative (fibrinopurulent, purulent and/or hemorrhagic) salpingitis, with/without congestion, and/or exudative peritonitis and/or exudative oophoritis. Recorded as acute if no or very little exudate or chronic if large accumulations of exudate
	Chronic salpingitis	Salpingitis in a chronic stage with large accumulations of inflammatory exudate in the oviduct lumen
	Septicemia	Focal necrosis of liver and/or spleen, hepato- and/or splenomegaly and/or endocarditis without other primary lesions (e.g. salpingitis) and/or if bacteria can be isolated from liver and/or spleen
	Arthritis or bumble foot	Arthritis or bumble foot, and sequel conditions: septicemia, nephropathy, and/or amyloidosis, or if observed in euthanized hens
	Polyserositis	Inflammation of multiple serous membranes, such as airsacculitis, perihepatitis and/or pericarditis
	Miscellaneous	Other infectious disorders, such as osteomyelitis, bursitis presternalis etc.
Non-infectious causes of mortality	Cannibalism	Primary cannibalism (ante mortem) with lesions at cloacal region with/without necrosis of mucosal membrane, presence of blood in cloacal region and/or in feathers and/or pale musculature and/or comb, and tissue loss (musculature, skin), or partial or full evisceration (oviduct, intestines etc.) with coagulated blood in the abdomen
	Egg bound	Vascular congestion in oviduct and/or ovary, possibly signs of congestion in other organs, and a fully developed egg in caudal part of the oviduct
	Pecking	Signs of severe injurious pecking (blood and/or necrosis present around lesion), signs of anemia. Toe pecking or other types of pecking (no tissue loss or evisceration)
	Uremia	Nephropathy (increased tubular pattern and/or renomegaly and/or urate in ureters and/or pale kidneys), possibly visceral gout, and no other primary lesion (e.g. salpingitis, arthritis, neoplasia, gastrointestinal disorders, etc.)
	Internal hemorrhage	Coagulated blood in the coelomic cavity, signs of anemia. Liver rupture or other origin
	Gastrointestinal disorders	Impactions of crop and/or intestines[a] and/or intestinal torsion and/or intussusception and/or invagination and/or pendulous crop and/or cloacal prolapse when not related to egg bound or pecking, with signs of circulatory collapse and/or emaciation
	Trauma	Fractures (with signs of hemorrhage) and/or bruises, signs of anemia
	Neoplasia	Diffuse or focal (solitary or multiple) cellular infiltrations in parenchymatous organs, ovary, oviduct, mesentery, etc.
	Cardiovascular disorders	Ventricular heart dilatation, heart failure/insufficiency, sudden death syndrome, unspecific generalized circulatory disturbances (without septicemia)
	Anemia	Primary anemia without signs of internal hemorrhage, cannibalism, pecking or trauma[b]
	Miscellaneous	Other non-infectious disorders, such as torsion of the oviduct etc.

[a] Impactions of straw material or excessive infestation of roundworms.
[b] Most often severe infestation of *Dermanyssus gallinae*.
